# Maternal Obesity as a Risk Factor for Caesarean Delivery in Sub-Saharan Africa: A Systematic Review

**DOI:** 10.3390/life12060906

**Published:** 2022-06-17

**Authors:** Jessica B. Brizan, Emmanuel Amabebe

**Affiliations:** 1 School of Health and Related Research, University of Sheffield, Sheffield S1 4DA, UK; jessica.brizan@gmail.com; 2 Department of Oncology and Metabolism, University of Sheffield, Sheffield S10 2SF, UK

**Keywords:** pregnancy, maternal obesity, caesarean delivery, sub-Saharan Africa, systematic review

## Abstract

**Background:** Maternal obesity is associated with several adverse reproductive outcomes. It is a growing public health burden in sub-Saharan Africa, a region with low resources and capacity to care for the large, affected population. **Objectives:** To assess the evidence of maternal obesity as a risk factor for caesarean delivery in women in sub-Saharan Africa. **Methods:** A systematic review of relevant original articles using PubMed, MEDLINE, and CINAHL was performed. Google Scholar and the reference lists of relevant systematic reviews and meta-analyses were also searched for other eligible studies. Observational studies assessing maternal body mass index (BMI) ≥ 30 kg/m^2^ before or during gestation and caesarean delivery as birth outcome were included. **Results:** All 17 studies were published between 2009 and 2021 and included 227,675 (236–153,102) participants. The prevalence of maternal obesity ranged from 3.9 to 44%. All except two studies (88%) indicated an association of obesity and risk of caesarean delivery in pregnant women in sub-Saharan Africa. Overweight/obese women had up to 4-fold increased risk of caesarean delivery compared to normal weight women. Three studies also reported a direct relationship between morbid obesity and prevalence of caesarean delivery in the sub-region. The risk of caesarean delivery appears to increase with increasing BMI e.g., >5 times in women with BMI ≥ 40 kg/m^2^ than in normal weight women. **Conclusions**: In sub-Saharan Africa, increased BMI in pregnancy is a risk factor for subsequent caesarean delivery. The risk of caesarean delivery appears to increase with increasing BMI. A robust meta-analysis and other patho-mechanistic studies can be conducted to confirm causal association. Culturally appropriate weight management and nutritional interventions should be implemented to reduce the incidence of obesity-induced caesarean delivery in sub-Saharan Africa.

## 1. Introduction

Obesity is a major global public health burden that contributes to more than 2 million preventable deaths every year [1,2]. It is an inflammatory disease [3] that is commonly defined as body mass index (BMI) ≥ 30 kg/m^2^ [4]. There is a higher prevalence of obesity in developed countries, however, it has become increasingly more prevalent in developing countries [5]. In Africa, obesity is often erroneously associated with high socioeconomic status (wealth) and health, particularly among women [6]. African women are about four times more likely to be obese than their male counterparts [7].

Pregnancy is a widely recognised catalyst for obesity in women [8]. Although maternal obesity is assessed differently worldwide, pre-pregnancy and first trimester BMI are the most commonly employed evaluation tools [9]. However, studies have reported maternal BMI at different gestational time points because in low resource settings, the first prenatal visit, when maternal BMI is commonly determined, often occurs after first trimester [10].

The prevalence of maternal obesity in Africa can be as high as 50% [11]. Older and multiparous women have a greater risk of being obese [11]. The prevalence of excessive gestational weight gain according to the American Institute of Medicine Gestational Weight Gain guidelines can also be as high as 37% [12]. There is also postpartum retention of excess weight gained during gestation with attendant risk of obesity later in life [13], and complications in the next pregnancy [14].

Gestational weight gain, which is sometimes induced by inflammation, is vital to the success of normal pregnancy [15]. The increase in BMI, especially at the later stages of gestation (i.e., third trimester), is believed to encourage energy storage in fat cells for rapid foetal growth and development, and in readiness for subsequent energy-demanding processes such as labour and lactation [15]. Despite this supposed beneficial effect of gestational weight gain [15], maternal obesity is associated with adverse reproductive and overall health outcomes and complications [16]. Significant adverse effects of maternal obesity on maternal, labour, and child outcomes in the African continent [11,17] as well as globally [14,18,19,20,21] have been reported. Overweight and obese women have increased risk of maternal complications including gestational hypertensive disorders, pre-eclampsia and eclampsia, gestational diabetes mellitus, induction of labour, prolonged labour, instrumental vaginal delivery, caesarean delivery, spontaneous and medically indicated preterm birth, miscarriage, postpartum haemorrhage, pulmonary embolism, genitourinary tract infection, postpartum weight retention, and maternal mortality. Their offsprings are also at increased risk of complications including congenital abnormalities, stillbirth, macrosomia (birthweight ≥ 4000 g), shoulder dystocia, respiratory distress syndrome, and intrauterine (foetal) and neonatal death [14,18,19,20,21,22,23,24,25,26,27,28]. In addition, both mother and infant are at a greater risk of developing subsequent non-communicable diseases such as obesity and cardiometabolic diseases later in life [18,19,22,24]. These complications can result in disabilities or mortalities especially in poor resource settings such as Africa where the capacity to manage such complications is limited [25]. 

The relationship between maternal obesity and delivery by caesarean section is well established in high resource settings [29,30], but less so in low income settings such as many countries in Africa [11]. Moreover, in Africa, studies assessing medical and obstetric interventions for obese pregnant women and evaluating the awareness or attitudes of pregnant women towards maternal obesity are scarce [11]. Therefore, in this systematic review, we investigated maternal obesity as a risk factor for caesarean delivery in adult women in sub-Saharan Africa. This was to specifically assess the relationship between maternal obesity and delivery by caesarean section in women in sub-Saharan Africa in comparison to pregnant women with normal or optimal BMI.

## 2. Materials and Methods 

### 2.1. Literature Search

We performed a systematic review according to the Preferred Reporting Items for Systematic Reviews and Meta-Analyses (PRISMA) guidelines. Relevant original articles were identified through a systematic search of PubMed, MEDLINE via Web of Science and CINAHL via EBSCO electronic databases without a date restriction, i.e., from the inception of the databases to April 2022. Google Scholar and the reference lists of relevant reviews and meta-analyses were also searched for other potentially eligible studies. The review question was broken down into concepts relating to the population, exposure, study design, and outcomes. The searches were conducted using a comprehensive search strategy focused on maternal obesity (exposure) and delivery by caesarean section (outcome). The search strategy was formulated by combining synonyms of the following key words and free text search terms: “maternal weight”, “caesarean delivery” and “Africa or sub-Saharan Africa” together with Boolean operators (OR/AND) to refine the search results. The search criteria from MEDLINE via Web of Science is presented in Table 1. Only peer-reviewed studies reported in English language were included. The process of study selection was based on the main inclusion and exclusion criteria employed, which are shown in Table 2. 

### 2.2. Study Selection

**Figure 1:**
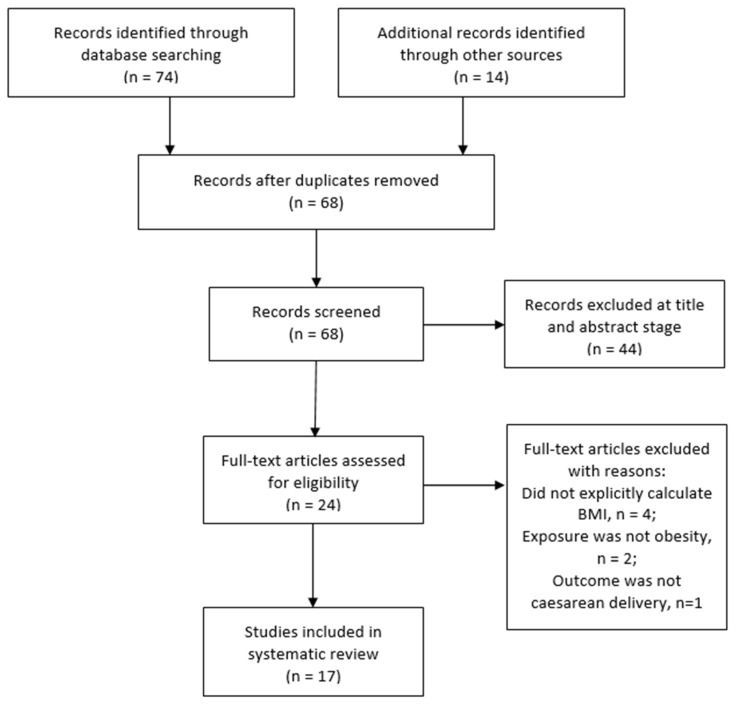
PRISMA flow chart of the study selection.

Using the inclusion criteria (Table 2), two review authors independently screened the titles and abstracts of studies retrieved to determine those that required further assessment. After duplicates were removed, the review authors further assessed the potential studies that were identified from the search strategy. When relevance could not be determined by title and abstract alone, the full-texts of the articles were retrieved and assessed for possible inclusion based on study design, type of participants, exposure and outcome measures. All studies that were excluded after assessment at the full-text stage were summarised with reasons for their exclusion in Supplementary Table S1. The stages of study selection were also presented on a PRISMA flowchart (Figure 1). 

### 2.3. Data Extraction and Quality Assessment

Data extraction was performed together by both authors using a data extraction form adapted from the Centre for Reviews and Dissemination (CRD) guidance. This form was used to summarise key details of each study that met the inclusion criteria. The included studies were further quality assessed by the two authors using the Critical Appraisals Skills Programme (CASP) Checklists for observational studies. These checklist criteria were modified to include assessment of cross-sectional studies. The following key details: study identifier, study characteristics, participant characteristics, exposure and setting, outcome data or results, and summary outcome data, were extracted from each study that met the inclusion criteria. A completed sample of the data extraction form and CASP Checklist can be found in Supplementary Tables S2 and S3, respectively.

### 2.4. Data Synthesis

In studies where they were reported, odds (ORs) and risk ratios (or relative risk, RR) as well as 95% Confidence Interval (CI) were calculated from the frequency (reported as percentage) or prevalence rates of caesarean delivery. If the unadjusted ORs and CIs were not reported, the respective adjusted parameters including *p* values were reported. The authors of the included studies were not contacted for any missing data. A narrative synthesis was used to summarise the findings of the studies included as it allowed for exploration of comparisons between the studies. Descriptive information about each study’s population, exposure, comparators, outcomes and measure of association were presented in text and tables and a forest plot. The country or countries where the included studies were conducted were noted, and the gestational age at which maternal obesity was measured was also recorded and described. Heterogeneity among included studies was assessed clinically and methodologically, and it was determined that a meta-analysis was not suitable for this data set due to the variation in study designs, methodology, and measurements of exposure between the studies.

## 3. Results

### 3.1. Literature Search

A total of 88 potentially relevant records (articles) were identified through the search process (Figure 1). The searches identified 40 records from MEDLINE, 18 from PubMed, and 16 from CINAHL. Fourteen additional records were identified through Google Scholar and the reference list of a systematic review and meta-analysis on maternal obesity in Africa [11]. Of the 88 total records, 20 were duplicates and were excluded. Sixty-eight records were screened by title and abstract, and 44 were excluded. Twenty-four full text articles were further screened for eligibility for inclusion in the review. After assessment, 7 studies [31,32,33,34,35,36,37] were excluded because they did not meet the inclusion criteria. The excluded studies and reasons for their exclusion are presented in Supplementary Table S1. The remaining 17 studies were included in this review and analysed further.

### 3.2. Study Characteristics

#### 3.2.1. Study Setting

Of the 17 studies, only 1 study used a pooled data set from 31 sub–Saharan African countries [38]. Most of the studies were conducted in Nigeria (6/17) [39,40,41,42,43,44]; and South Africa (3/17) [45,46,47] (Table 3). Twelve of the included studies were conducted either in tertiary health centres or maternity clinics [10,39,40,41,42,43,45,46,48,49,50,51]; while the other 5 studies were conducted in community or neighbourhood settings [38,44,47,52,53].

#### 3.2.2. Study Design

All the included studies were observational studies except for Davies et al., [47] which was a sub-study of a randomised controlled trial (Table 3). Three of the studies used a case-control design [39,40,45], six studies used a cross-sectional design [38,44,46,50,52,53], and seven studies used a cohort design [10,41,42,43,48,49,51].

### 3.3. Population

This review included studies comprising a total of 227,675 participants with sample sizes ranging from 236 to 153,102 (Table 3 and Table 4). BMI was recorded at various gestational time points across all the studies (from pre-pregnancy to 5 years post-delivery). Three studies recruited participants in the first trimester [40,43,51]. Seven studies recorded participants’ BMI at the first antenatal booking without stating a specific gestational age [40,41,45,46,47,48,50]. Minsart et al., [10] compared caesarean outcomes for BMI at ≤14 weeks, ≤22 weeks, and at delivery. Two other studies measured maternal BMI at ≤25 weeks [42] and ≤32 weeks [39]. Four cross-sectional studies accepted BMI measurements from participants who delivered up to 5 years before the study was conducted [38,44,52,53]. Only one retrospective study compared caesarean delivery outcomes in participants with maternal BMI recorded before pregnancy [49].

### 3.4. Types of Exposure

All studies reported BMI (kg/m^2^) as a measure of maternal obesity (Table 4). The maternal BMI exposure categories for overweight, obese, and morbidly obese varied according to the studies. Most studies used a BMI ≥ 30 kg/m^2^ as the cut-off for obesity except for Davies et al., [47], which used a BMI ≥ 29 kg/m^2^. Three studies combined overweight and obese participants and compared them to normal or optimal weight individuals (BMI = 18.5–24.9 kg/m^2^) [50,51,52]; while four other studies compared participants in different categories of obesity to those with normal/optimal weight [10,38,42,46]. 

### 3.5. Outcome Measure

**Figure 2:**
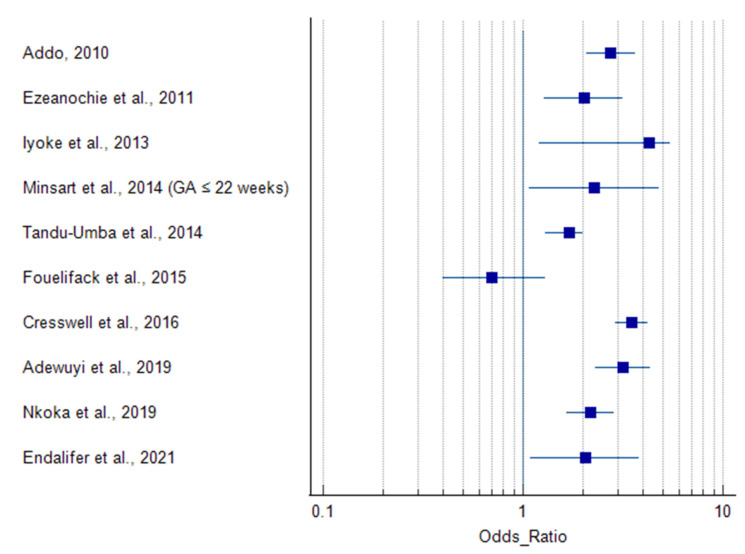
Summary results of the original articles showing that maternal obesity increases the risk of caesarean delivery. Only studies that reported odd ratios [10,38,40,43,44,49,50,51,52,53] were included in the forest plot. The results from studies without odds and risk ratios (*n* = 6) [39,41,42,45,46,47] and studies with sub-categories of caesarean delivery (*n* = 1) [48] were not included in the forest plot. Minsart et al., [10] recruited participants at two gestational time points but chose BMI ≤ 22 weeks as the main variable because it had a lesser amount of missing data compared to BMI ≤ 14 weeks, and BMI at first prenatal visit (which usually occurs after first trimester) is commonly used as the main variable regardless of gestational age [10].

All included studies reported delivery by caesarean section as an outcome measure (Table 2), along with several other maternal and neonatal outcomes and complications. The prevalence of obesity ranged from 3.9% to 44%. Delivery by caesarean section was associated with obesity in all of the studies except Basu et al., [46] and Fouelifack et al., [49], which did not identify an association (Table 4). Ugwuja et al., [42] (BMI > 35.0 kg/m^2^), Ngoga et al., [45] (BMI ≥ 40 kg/m^2^), and Cresswell et al., [38] (BMI > 35.0 and ≥ 40 kg/m^2^) reported an association between caesarean delivery and morbid obesity (Table 4). The prevalence of caesarean delivery in overweight and obese women was 1.5–4.3 times that of normal weight women. This margin rose up to 5.9 times in women with (BMI ≥ 40 kg/m^2^) [38]. Six studies did not report odds or risk ratios for caesarean delivery outcomes [39,41,42,45,46,47]. One study subdivided the delivery outcome into elective and emergency caesarean sections, which were both directly related to obesity [48]. These studies with missing and subdivided outcomes (*n* = 7) were not included in the forest plot (Figure 2).

### 3.6. Association of Maternal Obesity and Caesarean Delivery 

In 13 of the studies that had obese as a BMI category, 7 reported a significantly higher incidence/prevalence of caesarean delivery in obese women (BMI ≥ 30 kg/m^2^) compared to their non-obese counterparts. In two studies, there was no association between obesity and caesarean section [46,49]. However, in three other studies, morbidly obese women with a BMI > 35.0 kg/m^2^ [38,42] or BMI ≥ 40 kg/m^2^ [38,45], had significantly increased rates of caesarean delivery compared to non-obese women (20–25 kg/m^2^) (Table 4). Only one study categorized caesarean delivery into elective and emergency, and the obese women had greater risk of both elective [RR = 2.4 (1.88, 3.6)] and emergency [RR = 1.53 (1.34, 1.75)] caesarean sections [48]. In three studies, there was a significant increase in caesarean delivery in both overweight and obese women compared to non-obese women [50,51,52]. In the study that pooled results from 31 sub-Saharan African countries, women in each obesity class (i.e., Class I, II, and III) were more likely to have experienced a caesarean delivery compared to normal weight women [38]. Six studies did not report either an odds or risk ratio [39,41,42,45,46,47].

## 4. Discussion

This systematic review collated and assessed the available evidence of an association of maternal obesity and incidence of caesarean delivery in women in sub-Saharan Africa. All 17 included studies were published between 2009 and 2021 and included a total of 227,675 (236–153,102) participants. The prevalence of maternal obesity ranged from 3.9 to 44% across the studies. This wide range could be due to differences in study design, settings, and/or classification of obesity. Most (12/17) of the studies were conducted in healthcare facilities while 5 were community-based studies. All except two studies (88%) showed evidence of obesity as a risk factor for caesarean delivery in pregnant women in sub-Saharan Africa. That is, in sub-Saharan Africa, obesity significantly increases a woman’s risk of delivering by caesarean section. Overweight and obese women can have up to 4-fold increased risk of caesarean delivery compared to their normal weight counterparts. The risk of caesarean delivery appears to increase with increasing BMI. For instance, it is greater than 5 times in women with BMI ≥ 40 kg/m^2^ than in normal weight women. Most of the data (evidence, 7/17) were obtained from studies conducted in West Africa (i.e., Nigeria = 6, and Ghana = 1) within the last decade (2010–2019). 

Obesity, including maternal obesity, is a growing public health burden in sub-Saharan Africa, a region with low resources/capacity to care for the large, affected population liable to poor reproductive outcomes. While it focusses on sub-Saharan Africa, our study also validates and updates the findings of a previous meta-analysis on the effect of maternal obesity on six labour outcomes including caesarean delivery in Africa [11]. The meta-analysis reported that obese women were more likely to deliver by caesarean section than women who were not obese (RR 1.87, 1.64–2.12, *n* = 8) [11]. 

A clear dose-response relationship between maternal obesity and risk of caesarean delivery has been observed in sub-Saharan Africa [38]. That is, the likelihood of a woman delivering by caesarean section increases with an increasing BMI. For instance, morbidly obese (BMI ≥ 40 kg/m^2^) women can have more than a three-fold risk of delivering by caesarean section than normal weight women [38]. The prevalence of obesity in sub-Saharan Africa is projected to increase to 17.5% by 2030 [54]. With the increasing demand for caesarean section, which has already surpassed available capacity, pre- and post-pregnancy weight loss should be recommended for overweight and obese women of reproductive age in the sub-region [38,55].

We identified two studies [46,49] that did not indicate an association between maternal obesity and delivery by caesarean section. Although Basu et al., [46] did not identify an association between maternal obesity and delivery by caesarean section in their cohort, they recorded a high prevalence of obesity (44%), which was associated with other complications including gestational diabetes, urinary tract infection, and failed induction of labour. Similarly, Fouelifack et al., [49] did not observe a direct association between maternal obesity and delivery by caesarean section but reported that gestational weight gain above the American Institute of Medicine Gestational Weight Gain Recommendations [56] increased the risk of poor maternal outcomes including caesarean delivery, obstetrical haemorrhage or preeclampsia by 1.7-fold. The prevalence of maternal obesity was also relatively high (14%) compared to other studies.

Taken together, the findings of both studies corroborate the association of maternal obesity, gestational weight gain, adverse reproductive outcomes, and overall health complications in women, especially those living in the sub-region. The multiple comorbidities and complications associated with maternal obesity can be reduced if overweight, obesity, and excess gestational weight gain can be reduced in women of reproductive age [24]. Medical and obstetric management of obesity-associated complications during gestation, especially of blood glucose [14] and hypertension, should be accompanied with promotion of public health recommendations for a healthy diet and engaging in moderate physical exercise during pregnancy [12,21,28,57]. 

### Limitations

There are several limitations that may impact the interpretation and implications of the findings of this study. This study included mainly observational studies, some of which did not adjust for confounders. Those that did adjust for confounders differed by type and number of the confounding variables. Although we strictly followed a robust list of inclusion/exclusion criteria, we recognize that obesity may not have been the sole indication for caesarean delivery in the included studies. The BMI of the obese group in one of the studies [47], a sub-study from a RCT, may have also been misclassified. The included studies also measured BMI at different gestational time points, most of which were unspecified. Furthermore, there could be a difference in outcome between pre-pregnancy BMI and BMI measured during gestation. Generalisability of the findings is also impacted by the dominance of data from the West African sub-region compared to other sub-regions. There are 46 countries in sub-Saharan Africa (United Nations Development Programme) and only original articles from 8 countries in the sub-region were included in this study. The report from Demographic and Health Surveys pooled from 31 countries in the sub-region [38] could not ascertain the prevalence of elective vs. emergency caesarean sections, which could be impacted by BMI. Data extraction and quality assessment for this review was conducted by only two reviewers, therefore, an objective appraisal may not have been fully obtained. In addition, a meta-analysis on more homogenous studies could provide more precise evidence on the effect of maternal obesity on caesarean delivery. 

## 5. Conclusions

This review provides further evidence and validation that maternal obesity is a risk factor for caesarean section in sub-Saharan Africa. Obesity exerts significant influence on the mode of delivery, independent of other risk factors such as macrosomia, pre-eclampsia, and gestational diabetes, etc. The risk of caesarean delivery increases as BMI increases, plausibly more so for emergency caesarean section. Although this study has contributed more evidence to the limited literature on maternal obesity and its associated pregnancy outcomes in the African population, a robust meta-analysis and other patho-mechanistic studies can be conducted to confirm causal association. Culturally appropriate weight management and nutritional interventions should be implemented to reduce the incidence of obesity-induced caesarean delivery in sub-Saharan Africa.

## Figures and Tables

**Table 1:** Search strategy.

MEDLINE via Web of Science searched on 20 May 2020; updated on 25 April 2022
maternal weight or maternal overweight or maternal obesity or maternal bmi or gestational bmi or gestational weight or gestational obesity.mp caesarean section or caesarean outcome or caesarean delivery or caesarean birth.mp Africa or sub-Saharan Africa or Angola or Benin or Botswana or Burkina Faso or Burundi or Cameroon or Cape Verde or Central African Republic or Chad or Comoros or Congo Brazzaville or Democratic Republic of Congo or Cote d’Ivoire or Ivory Coast or Djibouti or Equatorial Guinea or Eritrea or Ethiopia or Gabon or The Gambia or Ghana or Guinea or Guinea-Bissau or Kenya or Lesotho or Liberia or Madagascar or Malawi or Mali or Mauritania or Mauritius or Mozambique or Namibia or Niger or Nigeria or Rwanda or Sao Tome or Principe or Senegal or Seychelles or Sierra Leone or Somalia or South Africa or Sudan or South Sudan or Swaziland or Eswatini or Tanzania or Togo or Uganda or Western Sahara or Zambia or Zimbabwe.mp 1 and 2 and 3

**Table 2:** Inclusion and exclusion criteria for study selection.

	Inclusion Criteria	Exclusion Criteria
**Population**	Pregnant women (mean/median age ≥ 18 years) living in sub-Saharan African countries	Adult women with specific disease conditions such as HIV infection, COVID-19, uterine haemorrhage, mastitis, uterine abnormality and infectious pregnancy complications, infant new-born diseases and multiple gestation
**Exposure**	BMI ≥ 30 kg/m^2^	Interventions to increase maternal weight, episiotomy, symphysiotomy, spinal anaesthesia, and hysterectomy
**Comparator**	BMI < 30 kg/m^2^ (normal weight or a combination of overweight and normal weight participants)	Studies that did not measure BMI
**Outcomes**	Delivery of a live neonate by caesarean section.	Studies that did not report caesarean delivery as an outcome
**Study type**	Observational studies such as case-control studies, cross-sectional studies, and cohort studies	Randomised controlled trials, studies on evaluation of health care, non-English language studies

*BMI*, body mass index; *HIV*, human immunodeficiency virus.

**Table 3:** Characteristics of the included studies.

Study	Country	Study Design	Population	BMI, kg/m^2^ Categories	Gestational Age
Ngoga et al., 2009 [45]	South Africa	Case-control	309	Morbidly obese (≥40)	Antenatal booking
Addo, 2010 [51]	Ghana	Retrospective cohort	1755	Overweight + Obese (≥25.1) *	First trimester
Basu et al., 2010 [46]	South Africa	Retrospective cross-sectional	767	Obese (30–39.9); Morbidly obese (≥40)	Antenatal booking
Adesina et al., 2011 [39]	Nigeria	Case-control	236	Obese (≥30)	≤32 weeks
Ezeanochie et al., 2011 [40]	Nigeria	Case-control	402	Obese (≥30)	First trimester
Israel et al., 2011 [41]	Nigeria	Prospective cohort	300	Obese (≥30)	Antenatal booking
Ugwuja et al., 2011 [42]	Nigeria	Prospective cohort	349	Obese (30–34.9); Morbidly obese (>35.0)	≤25 weeks
Davies et al., 2013 [47]	South Africa	RCT sub-study	1058	Obese (≥29)	Antenatal booking
Iyoke et al., 2013 [43]	Nigeria	Retrospective cohort	648	Obese (≥30)	First trimester
Minsart et al., 2014 [10]	Djibouti	Prospective cohort	497	Obese Class I: (30.0–34.9) Obese Class II: (≥35)	≤14 weeks ≤22 weeks At delivery
Tandu-Umba et al., 2014 [50]	Democratic Republic of Congo	Cross-sectional	2086	Overweight + Obese (≥28) *	Booking
Fouelifack et al., 2015 [49]	Cameroon	Retrospective cohort	462	Obese (≥30)	Pre-pregnancy
Cresswell et al., 2016 [38]	31 sub- Saharan African countries †	Pooled cross-sectional	153,102	Obese Class I: (30.0–34.9) Obese Class II: (35–39.9)Obese Class III: (≥40)	Up to 5 years post delivery
Adewuyi et al., 2019 [44]	Nigeria	Cross-sectional	31,171	Obese (≥30)	Up to 5 years post delivery
Nkoka et al., 2019 [53]	Malawi	Cross-sectional	15,732	Obese (≥30)	Up to 5 years post delivery
Mwanamsangu et al., 2020 [48]	Tanzania	Retrospective cohort	11,873	Obese (≥30)	First antenatal care visit
Endalifer et al., 2021 [52]	Ethiopia	Cross-sectional	6928	Overweight + Obese (≥25) *	Up to 5 years post delivery

* Studies that combined the obese (BMI ≥ 30 kg/m^2^) and overweight women (BMI ≥ 25 kg/m^2^). *BMI*, body mass index; *RCT*, randomised control trial. † Benin, Burkina Faso, Burundi, Cameroon, Chad, Comoros, Congo-Brazzaville, Cote d’Ivoire, Democratic Republic of Congo, Ethiopia, Gabon, Ghana, Guinea, Kenya, Lesotho, Liberia, Madagascar, Malawi, Mali, Mozambique, Namibia, Niger, Nigeria, Rwanda, Senegal, Sierra Leone, Swaziland, Tanzania, Uganda, Zambia, Zimbabwe.

**Table 4:** Summary results of included studies showing the prevalence of obesity and association between maternal obesity and caesarean delivery.

Study	Sample Size (*n*)	Obesity Prevalence (%)	Maternal Obesity Exposure (BMI, kg/m^2^)	Caesarean Delivery Outcomes OR/RR (95% CI)	*p*-Value
Ngoga et al., 2009 [45]	309	*NA*	Morbidly obese (≥40)	-	<0.001
Addo, 2010 [51]	1755	17.9 (314/1755)	Overweight + Obese (≥25.1)	OR: 2.74 (2.07, 3.64)	<0.01
Basu et al., 2010 [46]	767	44 (337/767)	Obese (30–39.9) Morbidly obese (≥40)	-	0.15
Adesina et al., 2011 [39]	236	*NA*	Obese (≥30)	-	0.00
Ezeanochie et al., 2011 [40]	402	9.6 ^a^ (201/2086)	Obese (≥30)	OR: 2.01 (1.27, 3.18)	0.003
Israel et al., 2011 [41]	300	7.4 ^b^ (357/4832)	Obese (≥30)	-	<0.001
Ugwuja et al., 2011 [42]	349	17.2 (60/349) 5.4 (19/349)	Obese (30–34.9) Morbidly obese (>35.0)	-	Not significant <0.05
Davies et al., 2013 [47]	1058	33.1 (350/1058)	Obese (≥29)	-	<0.001
Iyoke et al., 2013 [43]	648	17.9 ^c^ (340/1806)	Obese (≥30)	OR: 4.3 (1.2, 5.44)	<0.001
Minsart et al., 2014 [10]	497	25.2 ^d^ (112/445)	Obese (≥30)	GA ≤ 22 weeks: aOR: 2.27 (1.07, 4.82)	0.0321
Tandu-Umba et al., 2014 [50]	2086	21.9 (456/2086)	Overweight + Obese (≥28)	OR: 1.7 (1.3, 2.0)	<0.05
Fouelifack et al., 2015 [49]	462	14 (65/462)	Obese (≥30)	aOR: 0.7 (0.4, 1.3)	NS
Cresswell et al., 2016 [38]	153,102	3.9 (5970/153,102) 1.0 (1531/153,102) 0.4 (612/153,102)	Obese Class I: (30.0–34.9) Obese Class II: (35–39.9) Obese Class III: (≥40)	aOR: 3.50 (2.89, 4.24) aOR: 3.76 (2.71, 5.22) aOR: 5.90 (3.82, 9.11)	0.001
Adewuyi et al., 2019 [44]	31,171	8 (2469/31,171)	Obese (≥30)	aOR: 3.16 (2.30, 4.32)	<0.001
Nkoka et al., 2019 [53]	15,732	4.1 (643/15,732)	Obese (≥30)	aOR: 2.16 (1.65, 2.84)	<0.0001
Mwanamsangu et al., 2020 [48]	11,873	12.3 (1464/11,873)	Obese (≥30)	Elective CS: RR = 2.4 (1.88, 3.6) Emergency CS: RR = 1.53 (1.34, 1.75)	<0.001
Endalifer et al., 2021 [52]	6928	10 (692/6928)	Overweight + Obese (≥25)	aOR: 2.05 (1.09, 3.83)	0.033

^a^ Prevalence of obesity was calculated from 201 obese women in the total number of eligible women that presented for antenatal care within the study period (*n* = 2086). ^b^ Prevalence of obesity was calculated from 357 obese women in the total number of women who registered for antenatal care (*n* = 4832). ^c^ Prevalence of obesity was calculated from 340 obese women in the total number of women who presented for antenatal care at the first trimester (*n* = 1806). ^d^ Prevalence of obesity was calculated from 112 obese women in the total number of women included in the study with BMI data (*n* = 445). Women with missing data *n* = 52 were excluded. The case-control studies [39,45] did not report prevalence of obesity, and hence, designated *NA*. Six studies did not report either an odds or risk ratio [39,41,42,45,46,47]. *BMI*, body mass index; *CI*, confidence interval; *CS*, caesarean section; *RR*, risk ratio (or relative risk); *aOR*, adjusted odds ratio; *OR*, odds ratio; *GA*, gestational age; *NA*, not applicable; *NS*, Not significant.

## Data Availability

Not applicable.

## Supplementary Materials

The following are available online at https://www.mdpi.com/article/10.3390/life12060906/s1, Table S1: Full-text articles excluded with reasons. Table S2: Sample of data extraction form. Table S3: Sample of quality assessment checklist.
