# Supplementary material for: Maternal Obesity as a Risk Factor for Caesarean Delivery in Sub-Saharan Africa: A Systematic Review
Source: Life (Basel). 2022 Jun 17;12(6):906. doi: 10.3390/life12060906 (PMC9229092; doi:10.3390/life12060906)
Supplement: Supplementary file 1 [file life-12-00906-s001.zip › life-1753044-supplementary.pdf]

Supplementary Material

Supplementary Table S1: Full-text articles excluded with reasons

Excluded studies	Reason for exclusion
Olayemi et al., 2002 [31]	BMI was not measured
Puotege et al., 2020 [32]	The exposure of the study was weight gain during pregnancy instead of obesity
Akinola et al., 2014 [33]	BMI was not measured
Edomwonyi & Osaigbovo 2006 [34]	Outcome was obesity incidence not caesarean delivery
Efiong 1975 [35]	BMI was not measured
Moraes et al., [36]	Maternal age was the exposure compared
Mpotora et al., 2021 [37]	BMI was not measured

Supplementary Table S2: Sample of data extraction form

**General information**	**Details**
Author, Year	Adesina et al, 2011
Article title	Pregnancy outcome of the obese in Ilorin
Country of origin	Nigeria
**Study characteristics**	
Study design	Case- control
Study inclusion and exclusion criteria	Gestational age less than 32 weeks
Recruitment procedures used	Booked pregnant women attending the antenatal clinic of the University of Ilorin Teaching Hospital
Unit of allocation	Participant
**Participant characteristics**	
Age	20 +
Disease characteristics	BMI greater than 30kg/m2
Number of participants enrolled	236
Number of withdrawals, exclusions, lost to follow-up	N/A
Number of participants in each characteristic category	Obese: 80 Non-obese: 156
**Exposure and setting**	
Setting in which the intervention is delivered	Hospital antenatal care
Description of the exposure and control	Exposure: obese (BMI greater than 30 kg/m2)<br>Control: not obese (BMI less than 30 kg/m2)
**Outcome data/results**	
Unit of assessment/analysis	Frequency of caesarean delivery as percentage
Statistical techniques used	Chi squared test ($\chi^2 = 9.87$, P = 0.00)
**Summary outcome data**	
Number of caesarean deliveries/ number of obese	63/156
Type of analysis used in study	Odds ratio, confidence intervals
**Results of study analysis**	1.91 (0.99-3.66)

Supplementary Table S3: Sample of quality assessment checklist
Critical Appraisal Skills Programme (CASP) Checklist for Adesina et al., [39]

Question	Answer	Comments
1. Did the study address a clearly focused issue?	Yes	Study focused on the effects of obesity on obstetric performance.
2. Did the authors use an appropriate method to answer their question?	Yes	A case control study design is an appropriate method.
3. Were the participants recruited in an acceptable way?	Yes	Participants were selected by BMI and gestational age. Cases were matched for age, parity and gestational age.
4. Was the exposure accurately measured to minimise bias?	Yes	Obesity was measured by BMI.
5. Was the outcome accurately measured to minimise bias?	Yes	The outcome was occurrence of caesarean delivery.
6. Have the authors taken account of the potential confounding factors in the design and/or in their analysis?	Yes	Cases and controls were matched.
7. How large was the exposure effect?	Can't Tell	Study used chi square to determine if there was an association.
8. How precise was the estimate of the exposure effect?	Can't Tell	P-value was used.
9. Do you believe the results?	Yes	Obese women had increased risk of caesarean delivery.
10. Can the results be applied to the local population?	Can't Tell	
11. Do the results of this study fit with other available evidence?	Yes	The author cited comparable findings.
